# Water Cycle Bat Algorithm and Dictionary-Based Deformable Model for Lung Tumor Segmentation

**DOI:** 10.1155/2021/3492099

**Published:** 2021-11-22

**Authors:** Mamtha V. Shetty, D. Jayadevappa, G. N. Veena

**Affiliations:** ^1^JSS Academy of Technical Education, Bengaluru, VTU, India; ^2^Ramaiah Institute of Technology, Bengaluru, India

## Abstract

Among the different types of cancers, lung cancer is one of the widespread diseases which causes the highest number of deaths every year. The early detection of lung cancer is very essential for increasing the survival rate in patients. Although computed tomography (CT) is the preferred choice for lungs imaging, sometimes CT images may produce less tumor visibility regions and unconstructive rates in tumor portions. Hence, the development of an efficient segmentation technique is necessary. In this paper, water cycle bat algorithm- (WCBA-) based deformable model approach is proposed for lung tumor segmentation. In the preprocessing stage, a median filter is used to remove the noise from the input image and to segment the lung lobe regions, and Bayesian fuzzy clustering is applied. In the proposed method, deformable model is modified by the dictionary-based algorithm to segment the lung tumor accurately. In the dictionary-based algorithm, the update equation is modified by the proposed WCBA and is designed by integrating water cycle algorithm (WCA) and bat algorithm (BA).

## 1. Introduction

Lung cancer is considered as the second most common kind of cancer for both male and females worldwide. As per the World Health Organization (WHO) statistics, 1.3 million deaths are happening because of lung cancer. Besides, it is calculated in the United States (US) that every year, approximately 228,820 people are newly affected by lung cancer in which 112,520 are women and 116,300 are men. Also, nearly 135,720 deaths are caused by lung cancer disease. The computer vision system has various tools, and these tools are used in different medical applications, especially for medical image analysis to diagnose various diseases [1, 2]. Computed tomography (CT) is a basic imaging modality which effectively helps for the detection of lung cancers. As per the statistics, lung cancer is the fourth major cause of death globally. The initial process of lung cancer detection is manual detection of lung regions in CT images by specialists, which is a more challenging and tedious process for computer vision models. The number of deaths due to lung cancer can be considerably decreased, when the lung CT screening is effective. However, it is a challenging process for radiologists to make effective and precise detection for large scale of CT images. Hence, automated segmentation techniques are introduced to overcome these difficulties. In addition, end-to-end probabilistic detection model was developed based on deep three dimensional convolutional neural networks (CNNs) to overcome uncertainty complexities [3]. Lung cancer is a malignant tumor, which is developed due to the abnormal development of cells in lung regions. The early diagnosis of lung cancer can drastically reduce the death rate, and survival rate of patients can be improved. Therefore, the early prediction is essential for enhancing the clinical situations of patients; thereby, it is more crucial for developing an effective technique for early prediction of lung cancer. In fact, low dose CT is considered as a secure and effective tool for preventive detection of high risk populations [4]. Segmentation of CT images plays a very significant role in lung cancer detection [5]. However, there are few issues such as same image densities in scanning protocols and variations of pulmonary structures due to scanner. Most of the existing semiautomatic segmentation techniques depends on human factors, thereby affecting the segmentation accuracy. Recently, various lung segmentation techniques have been developed using deep learning architectures, which are presently applied for clinical applications. Among these, U-Net architecture offers better performance in deep neural networks.

Generally, radiologists frequently use computer-aided design (CAD) system in order to offer secondary consideration for more precise detection. Moreover, this system is more useful to enhance the efficiency of detection rate. In the literature, various approaches are available to perform medical image segmentation. But, they are unable to segment lung nodules which are connected to the lung walls. Therefore, deep learning techniques are more effective for such applications. Deep learning models are capable of identifying the significant features of medical images; thereby, major drawbacks of handcrafted features are resolved [6]. Semiautomated segmentation methods are also available for the lung tumor segmentation and lung cancer classification, which includes marker-controlled watershed technique [7] and single click ensemble model [8]. But automatic segmentation and classification approaches are more effective and accurate as compared semiautomatic methods [9, 10]. Furthermore, every image classification model needs a suitable object segmentation approach. Several researches are focused for enhancing effective classification of lung cancers using multiscale Gaussian filter based on active contour and CNN method [11] and also convolutional transfer neural network with modified U-Net structures.

The main intention of this research is to design the development and performance evaluation of lung cancer segmentation algorithm using WCBA based deformable model approach. Lung tumor segmentation is a challenging issue due to in-homogeneities in the lung regions. This reason motivated us to develop a new methodology for the effective segmentation of lung tumor using CT images.

## 2. Literature Review

The early detection of lung cancer is a challenging issue due to complex structure of cancer cells, where most of the cancer cells are overlapped each other. For early diagnosis and treatment are very important to reduce the death rate. Researchers have been working towards the development of system which can detect cancer in its early stage and also tried to improve the accuracy of the system by incorporating preprocessing, segmentation, feature extraction, and classification techniques. The significant contributions of the existing research work and their limitations are presented below.

Hu et al. [12] developed a mask region-based CNN approach for lung tumor segmentation. In this method, Region Proposal Network (RPN) was employed for detecting object cases. This approach achieved better classification accuracy, but still failed to reduce the segmentation time. Ozdemir et al. [13] designed a deep learning approach for lung cancer segmentation using CT images. Here, data augmentation process was performed for reducing the overfitting issues. This technique obtained effectual diagnostic interventions. However, this model failed to visually evaluate learned feature representations for better performance. Shakeel et al. [14] designed improved profuse clustering technique (IPCT) and deep learning scheme for lung cancer detection. In addition, a weighted mean histogram equalization model was applied for removing the noises from the input CT image. In this method, IPCT-based spectral super pixel clustering was employed for the segmentation process. This approach has minimum misclassification error, although computational difficulty is high. Suresh and Mohan [15] presented CNN architecture for detecting lung cancer. In this technique, preprocessing was carried out to eliminate noise and desired nodule region is extracted. In addition, generative adversarial network (GAN) was applied for generating similar character images. This model enhanced the prediction speed, even though it is failed to explore optimal size of input patch in order to enhance the performance.

Jiang et al. [16] modeled multiple resolution residually connected network (MRRN) for lung tumor segmentation using CT images. This method resulted better segmentation accuracy independent of tumor location and size, but still this approach failed to reduce the computational complexity. Yu et al. [17] devised adaptive hierarchical heuristic mathematical model (AHHMM) for lung cancer detection using CT images. Here, weighted mean histogram approach was applied for removing of noise from input and enhancing the image quality. Then, *K*-means clustering was applied in order to obtain segmented tumor regions. Finally, a deep learning is introduced for predicting lung cancer. Singadkar et al. [18] developed deep deconvolutional residual network (DDRN) for lung nodule segmentation using CT images. Also, summation-based long skip connection from the network was applied for conserving spatial information. This technique obtained more precise classification of nodules in lung cancer. However, it is failed to enhance pulmonary nodule segmentation accuracy for other kinds of nodules. Jalali et al. [19] introduced deep neural network (DNN) for lung cancer segmentation. Here, morphological function was applied for extracting ground truth images. Additionally, bidirectional convolutional long short term memory (CLSTM) and ResNet-34 network, termed Res BCDU-Net, were introduced for effective training. The execution time of this approach was less; however, this model failed to integrate deep learning-based methods for obtaining better segmentation results.

The mask region-based CNN approach was developed for lung tumor segmentation using CT images. But this model was failed to provide accurate classification. The deep learning architecture was devised for the classification of lung cancer. But, this technique was failed to integrate decline choice and patient referral during the training process for better performance. The CNN architecture was used for detecting lung cancer, but this approach failed to produce high-quality realistic fake lung nodule samples to enhance detection performance in cancer images. The DNN approach was applied for the lung cancer segmentation process, but is also failed to perform the testing process for obtaining effectual segmentation output.

## 3. Proposed Methodology

**Figure 1:**
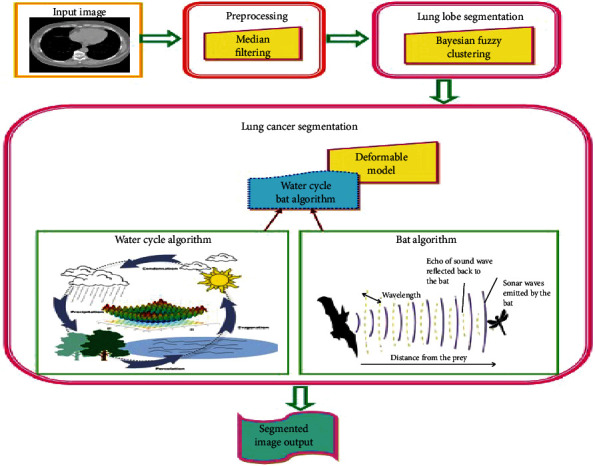
Block diagram of the proposed WCBA-based lung cancer segmentation model.

The proposed WCBA-based deformable approach for lung cancer segmentation model mainly includes three stages; preprocessing, lung lobe segmentation, and lung cancer segmentation. In the first stage, preprocessing of the input image is carried out using median filter to remove the noise. In the second stage, the lung lobe regions are segmented using Bayesian fuzzy clustering [20]. Finally, segmentation of lung cancer is performed using deformable model which was modified by dictionary-based algorithm [21]. Here, the equation is updated by the proposed WCBA optimization algorithm. This algorithm is designed by combining BA [22] and WCA [23]. The block diagram of the WCBA-based deformable model for lung cancer segmentation is depicted in Figure 1.

Let us assume a database *H* with *r* number of images, which is expressed as

(1) $$\begin{array}{c} H=\left\{T_{1},T_{2},\cdots T_{n},\cdots T_{r}\right\}, \end{array}$$

where *T*_*n*_ denotes *n*^th^ image in database, and *r* is a total amount of image in dataset. Here, the input image *T*_*n*_ is subjected to preprocessing to remove noise. The expression for the filtered image is as follows.

(2) $$\begin{array}{c} P\left(u,v\right)={\text{median}}_{\left(Q_{1}\right)\in {Χ}_{u,v}}\left\{f\left(Q_{1}\right.\right\}, \end{array}$$

where *u* and *v* are coordinates of pixel positions. The median filter output is represented as *Z*_1_.

### 3.1. Lung Lobe Segmentation Using Bayesian Fuzzy Clustering (BFC)

The preprocessed output *Z*_1_ is taken as input for lung lobe segmentation. The estimation quantity is effectively increased with less computational complexity in Bayesian fuzzy clustering (BFC) approach. Thus, the BFC technique is employed for segmenting lung lobe regions. This model combines joint likelihood function for segmenting lone regions. The core and edema tumor sections are identified by preserving edge and texture. Here, the BFC technique segments the input by prototype clustering process. Moreover, BFC uses membership function *F* and even symmetric Dirichlet proposal *q* for finding cluster prototypes, which is specified as

(3) $$\begin{array}{c} q_{w}^{+}\sim \text{Dirichlet }\left(k=1_{o}\right), \end{array}$$

where *q*_*w*_^+^ indicates regular symmetric Dirichlet proposal, and *O* refers total clusters in segmentation process. In addition, BFC uses conditional distribution with membership function *F* in order to estimate cluster prototype. The conditional distribution employed in BFC is given as

(4) $$\begin{array}{c} \tilde{Τ}\left({Χ}_{s}^{m},q_{w}\left|Y\right.\right)=Τ\left({Χ}_{s}^{m}\left|q_{w}\right.,D\right)\tilde{Τ}\left(\left|q_{w}\right.Y\right),\\ \tilde{Τ}\left({Χ}_{s}^{m},q_{w}\left|Y\right.\right)=\overset{Ο}{\underset{j=1}{\sum }}\tau \left({Χ}_{s}^{m}\left|t_{w}\right.,q_{wj}^{-Y}\right)q_{wj}^{-Y\tau /2}\text{Dirichlet}\left(t_{s}\left|\varpi \right.\right), \end{array}$$

where *Y* indicates membership function, and *t*_*w*_ is maximum-a posteriori (MAP) sample. The membership function utilized for estimating cluster prototypes is varied due to Gaussian distribution properties, thus, Markov chain state rule is used to get cluster prototype, which is specified as

(5) $$\begin{array}{c} T\left(X_{s}^{m},q_{w}\left|Y\right.\right)=T\left(X_{s}^{m}\left|D\right.,q_{w}\right)T\left(q_{w}\right). \end{array}$$

The likelihood value of cluster prototype is identified, and cluster prototype with enhanced likelihood value is taken as final segmented output. Hence, BFC technique predicts *O* segments for image slice *X*_*s*_^*m*^ is specified as

(6) $$\begin{array}{c} P=\left\{P_{1}^{s,m},P_{2}^{s,m},\cdots ,P_{w}^{s,m},\cdots ,P_{o}^{s,m}\right\}, \end{array}$$

where *P*_*w*_^*s*,*m*^ indicates *w*^th^ image segment *X*_*s*_^*m*^. The output of lung lobe segmentation process is represented as *R*_*x*_, and it is given to lung cancer segmentation.

### 3.2. Lung Cancer Segmentation Using Water Cycle Bat Algorithm- (WCBA-) Based Deformable Model

The segmented lung lobe regions *R*_*s*_ from preprocessed image are considered as input for lung cancer segmentation. Here, the deformable model is developed for segmenting the lung cancer. Generally, deformable model is a type of surfaces or curves defined in image, which can move under the pressure of internal forces. In addition, the deformable model is a geometric pattern, and its shape varies depending on time. Thus, in this technique, deformable approach is introduced by the modification dictionary-based image segmentation. Meanwhile, in dictionary-based image segmentation, the equation is updated based on the proposed WCBA optimization technique and is designed by integrating WCA and BA models.

Bat algorithm is devised by the inspiration of echolocation feature of microbats. This model is very effective to create improved features for solving multiobjective optimization problems. In addition, it has better capacity for solving high nonlinear problems with complex restrictions. On the other hand, WCA is motivated by nature, and it is devised by water cycle observation, river over sea, and stream flow in real-time situation. Moreover, WCA has the ability to solve various engineering design and managed optimization problems. WCA is effective to offer qualitative solutions and successful computational effectiveness. This method is used to address real-time optimization complexities with sufficient accuracy. In addition, it is effective for controlling dissimilar combinatorial optimization problems and affords optimal solution with less computational difficulty. Thus, the BA is integrated with WCA in order to obtain effective performance for lung cancer segmentation. The algorithmic steps for the proposed method are as follows.

**Step 1 .**

Initialization.

Initially, populations of raindrops are randomly created, which is expressed as

(7) $$\begin{array}{c} L\left\{L_{1},L_{2},.\cdots ,L_{k},\cdots L_{f}\right\};1\leq k\leq f, \end{array}$$

where *f* represents total number of raindrops population, and *L*_*k*_ specifies *v*^th^ population.

**Step 2 .**

Fitness function estimation.

The fitness measure is estimated in order to predict the optimum solution for effectual lung cancer segmentation process. The fitness function with the least value is considered as the best solution, and it is estimated by equation (26), which is specified as minimization function.

**Step 3 .**

Calculate each raindrop value.

After the computation of fitness value, the cost of all raindrops is computed by below equation

(8) $$\begin{array}{c} B_{y}=f\left(L_{1}^{y},L_{2}^{y},\cdots L_{f\mathrm{var}}^{y}\right);y=1,2,\cdots f_{pop}, \end{array}$$

where *B*_*y*_ denotes the cost of raindrop, *f*_*pop*_ is the amount of raindrops, and *f*_var_ is the number of design variables.

**Step 4 .**

Calculation of flow intensity for sea and rivers.

The flow intensity of river and sea is estimated in order to allocate raindrops, which is calculated as

(9) $$\begin{array}{c} fH=\text{round}\left(\left|\frac{B_{i}}{\sum_{o=1}^{f_{m}}B_{i}}\right|{Xf}_{\text{raindrops}}\right);i=1,2,\cdots f_{m}, \end{array}$$

where *fH*_*i*_ is number of streams, which flow to particular sea or rivers.

**Step 5 .**

Stream flow to river.

After the computation of flow intensity for river and sea, then streams flow to rivers is estimated by

(10) $$\begin{array}{c} L_{\text{stream}}^{b+1}=L_{\text{stream}}^{b}+\mathrm{rand}z\left(L_{\text{river}}^{b}-L_{\text{stream}}^{b}\right). \end{array}$$

**Step 6 .**

River flow to sea.

Here, stream flow to river is estimated in which solution update is performed by developed WSBA. In this stage, rivers flow to sea is estimated by

(11) $$\begin{array}{c} L_{\text{river}}^{b+1}=L_{\text{river}}^{b}+\mathrm{rand}z\left(L_{\text{sea}}^{b}-L_{\text{river}}^{b}\right). \end{array}$$

(12) $$\begin{array}{c} L_{\text{river}}^{b+1}=L_{\text{river}}^{b}\left(1-\mathrm{rand}z\right)+\left.\mathrm{rand}{zL}_{\text{sea}}^{b}\right). \end{array}$$

Moreover, the standard position update equation of BA is

(13) $$\begin{array}{c} L_{a}^{b}=L_{b}^{a-1}+Q_{b}^{a},\\ L_{a}^{b}=L_{b}^{a-1}+Q_{b}^{a-1}+\left(L_{b}^{a}-L_{s}\right)l_{b}. \end{array}$$

Moreover, it is expressed in terms of WCA,

(14) $$\begin{array}{c} L_{\text{river}}^{b}=L_{\text{river}}^{b-1}+Q^{b-1}+\left({Lb}_{\text{river}}^{b}-L_{\text{sea}}\right)l_{b}, \end{array}$$

(15) $$\begin{array}{c} L_{\text{river}}^{b}=L_{\text{river}}^{b-1}+Q^{b-1}+L_{\text{river}}^{b}l_{b}-L_{\text{sea}}l_{b}, \end{array}$$

(16) $$\begin{array}{c} L_{\text{sea}}=\frac{L_{\text{river}}^{b-1}+Q^{b-1}+L_{\text{river}}^{b}l_{b}-L_{\text{river}}^{b}}{l_{b}}, \end{array}$$

(17) $$\begin{array}{c} L_{\text{sea}}=\frac{L_{\text{river}}^{b-1}+Q^{b-1}+L_{\text{river}}^{b}\left(l_{b}-1\right)}{l_{b}}. \end{array}$$

Substituting equation (17) in (12),

(18) $$\begin{array}{c} L_{\text{river}}^{b+1}=L_{\text{river}}^{b}\left(1-\mathrm{rand}z\right)+\mathrm{rand}z\left(\frac{L_{\text{river}}^{b-1}+Q^{b-1}+L_{\text{river}}^{b}\left(l_{b}-1\right)}{l_{b}}\right), \end{array}$$

(19) $$\begin{array}{c} L_{\text{river}}^{b+1}=L_{\text{river}}^{b}\left(1-\mathrm{rand}z\right)+\frac{\mathrm{rand}z\left(l_{b}-1\right)L_{\text{river}}^{b}}{\;l_{b}}+\mathrm{rand}z\left(L_{\text{river}}^{b-1}+Q^{b-1}\right), \end{array}$$

(20) $$\begin{array}{c} L_{\text{river}}^{b+1}=L_{\text{river}}^{b}\left(\frac{1-\mathrm{rand}z+\mathrm{rand}{zl}_{b}-\mathrm{rand}z}{l_{b}}\right)+\mathrm{rand}z\left(L_{\text{river}}^{b-1}+Q^{b-1}\right), \end{array}$$

(21) $$\begin{array}{c} L_{\text{river}}^{b+1}=L_{\text{river}}^{b}\left(\frac{1-\mathrm{rand}z\left(2-l_{b}\right)}{l_{b}}\right)+\mathrm{rand}z\left(L_{\text{river}}^{b-1}+Q^{b-1}\right). \end{array}$$

Thus, the above expression defines the final updated equation for the developed WSBA.

Where rand specifies uniformly distributed random integer, which ranges between [0, 1], *l*_*b*_ = *l*_min_ + (*l*_max_ − *l*_min_)*k*, *l*_min_ = 0, *l*_max_ = 100, and *k* = [0, 1], *z* ranges from 1 to 1, *Q* denotes velocity, and *L*_river_^*b*^ signifies the position of river at *b*^th^ iteration.

**Step 7 .**

Replace river location.

Here, river position is replaced with stream, and it affords better solution. Moreover, if a river identifies the best solution than sea, then, location of river is replaced with sea.

**Step 8 .**

Evaporation circumstance.

Evaporation is a most important factor, which avoids rapid convergence, and evaporation circumstance, which is given by

(22) $$\begin{array}{c} \left|L_{\text{sea}}^{b}-\right|L_{\text{sea}}^{b}\left|L_{\text{sea}}^{b}-L_{\text{river}}^{b}\right|<B_{\max};b=1,2,3,\cdots f_{m}-1. \end{array}$$

If above equation is satisfied, then, raining procedure is executed where *B*_max_ is a small integer, which is near to zero.

**Step 9 .**

Raining process.

Once the evaporation condition is satisfied, then, raining process is done. The new position of newly generated streams is located by

(23) $$\begin{array}{c} L_{\text{stream}}^{\text{new}}=\text{LB}+\mathrm{rand}\left(\text{UB}‐\text{LB}\right), \end{array}$$

where LB and UB indicate lower bound and upper bound.

Moreover, computational performance and convergence rate are improved by the following equation, and it is only utilized for stream, which is directly flow to sea.

(24) $$\begin{array}{c} L_{\text{stream}}^{\text{new}}=L_{\text{sea}}+\sqrt{\delta \mathrm{rand}a}\left(1,f_{\mathrm{var}}\right), \end{array}$$

where *δ* denotes a coefficient, and it shows the range of searching area near sea, rand*a* is randomly distributed integer.

**Step 10 .**

Decrease value of user define parameter.

The large amount of *B*_max_ decreases a search, but minimum value supports search intensity near a sea. The value of *B*_max_ is decreased by

(25) $$\begin{array}{c} B_{\max}^{b+1}=B_{\max}^{b}-\frac{B_{\max}^{b}}{\max\text{iteration}}. \end{array}$$

**Step 11 .**

Check the feasibility of solution.

The optimal solution is estimated by fitness measure using equation (26) and if a new solution is better than previous solution then updates a previous value with new one.

### 3.3. Optimized Dictionary-Based Algorithm

The optimized dictionary-based image segmentation process is as follows.

#### 3.3.1. Region-Based Curve Evolution

Let us assume an image *J* with background and object, which is characterized by various two intensities. The curve is initialized in image, and it is specified by zero level set of function *ϕ*, which directs to pixel labeling as outside or inside. Moreover, mean label intensities *t*_out_ and *t*_in_ are computed in which outside includes more backgrounds, whereas inside involves more objects. Meanwhile, the curve is introduced for segmenting the image. Furthermore, curves are reduced, while pixel intensities are near to *t*_out_, and curves are enlarged, when pixel intensities are near to *t*_in_. The threshold value among enlargement and reduction is referred as (*t*_in_ + *t*_out_)(*t*_in_ + *t*_out_)/2, and this two-stage process is continued. The mean label intensities are recomputed in order to differentiate intensities of background and object, since curve is developed, and at last curve segments out the object. The zero level set evolution is specified by

(26) $$\begin{array}{c} \frac{\partial \varphi }{\partial t}=\eta_{\in }\left(\varphi \right)\left[\left(t_{\text{out}}-t_{\text{in}}\right)\left(2J-t_{\text{out}}-t_{\text{in}}+p_{h}\right)\right], \end{array}$$

where *p* specified minimizing curve length, and *h* denotes curvature of level set curve and term weighted, which is expressed as

(27) $$\begin{array}{c} p=\nabla \left(\frac{\nabla \varphi }{\left|\nabla \varphi \right|}\right). \end{array}$$

#### 3.3.2. Texture Dictionary

Here, particular amount of *TxT* patches is extracted from image and collects pixels intensities in patch vector of *t* = *T*^2^length and cluster are patches in *d*^th^ clusters based on *k*-means approach and Euclidean distance for creating dictionary. All image patches are allocated to single dictionary component and all image pixels related to *t* = *T*^2^ dictionary pixels in which the image patches are overlapping. Moreover, allocation of particular image patch to certain dictionary component produces *t* pixels from image patch relate to *t* pixels from dictionary part. In addition, binary relation among dictionary pixels and image pixels is specified based on sparse binary matrix *G* with |*φ*| rows and *dt* columns; here, |*φ*| defines the whole amount of image pixel, and *dt* refers to the whole quantity of dictionary pixels. Here, matrix *G* captures texture details of image by concurrently encoding two things, namely, spatial relationship among patches and dictionary task of all image patches.

#### 3.3.3. Label to Probability Transformation

Every patch from image *J* has equivalent label patch from *C*_in_. All dictionary units have the amount of allocated image patches, and it permits to estimate dictionary component label. Moreover, dictionary unit label is estimated as pixel-wise average of label patch related to image patches, which are allocated to dictionary unit. Dictionary label is effectively estimated by ordering label image pixels in binary vector *C*_in_ and multiplying with *G*, and it is normalized. The resulting vector includes pixel-wise frequency of dictionary unit belonging to the inside, which is expressed as

(28) $$\begin{array}{c} g_{\text{in}}=\mathrm{diag}{\left(GI\right)}^{-1}{Gc}_{\text{in}}. \end{array}$$

The elements of *g*_in_ is necessary to rearrange depending on dictionary dimension for obtaining dictionary label.

The labels in *g*_in_ and *g*_out_ = 1 − *g*_in_ are biased, because of the ratio of inside area |*φ*_in_| and outside area |*ψ*_out_ = |*ψ*| − |*ψ*_min_||. Moreover, pixel normalization function works on every element of *g*_in_, which is given by

(29) $$\begin{array}{c} {\tilde{g}}_{\text{in}}=\frac{1}{S}\frac{g_{\text{in}}}{\left|\psi_{\text{in}}\right|},\\ S=\frac{g_{\text{in}}}{\left|\psi_{\text{in}}\right|}+\frac{g_{\text{out}}}{\left|\psi_{\text{out}}\right|}. \end{array}$$

The next transformation includes the estimation of pixel-wise image probabilities from dictionary labels, which is carried out by averaging. Every dictionary label is located in image space at image patch location, which is allocated to dictionary unit. In order to estimate pixel probability, *t* values are required to average, because of patch overlap. Here, the effective estimated is done by

(30) $$\begin{array}{c} E_{\text{in}}=\mathrm{diag}{\left(G^{T}1\right)}^{-1}G^{T}{\tilde{g}}_{\text{in}}. \end{array}$$

Moreover, *E*_in_ is different from *C*_in_, because image patches from outside and inside is allocated to similar dictionary unit. The binary values from *C*_in_ is diffused depending on the texture information encoded in *G*.

#### 3.3.4. Multiple Labels

The layered image label with *C*_1_ to *C*_*x*_ layers is generated for managing multiple layers. In addition, every layer is a binary indicator of label, and the layers are sum to one in all pixels. The transformation is applied to every layers; thereby, dictionary labels are *g*_1_ to *g*_*x*_.. Meanwhile, normalization of area is carried out pixel-wise for every layer,

(31) $$\begin{array}{c} \left({\tilde{g}}_{1},{\tilde{g}}_{2},\cdots ,{\tilde{g}}_{Χ}\right)=\frac{1}{S}\left(\frac{g_{1}}{\left|\psi_{1}\right|},\frac{g_{2}}{\left|\psi_{21}\right|},\cdots ,\frac{g_{Χ}}{\left|\psi_{Χ}\right|}\right),\\ S=\overset{Χ}{\underset{x=1}{\sum }}\frac{g_{Χ}}{\left|\psi_{Χ}\right|}. \end{array}$$

Once area normalization is completed, then, transformation is applied to every ${\tilde{g}}_{Χ}$, thus, in *X* probability images *E*_1_ to *E*_*x*_, which is sum to one in every pixel.

#### 3.3.5. Curve Evolution

The closed curve is denoted as zero level set of function *ϕ*, which defines label image *C*_in_ attains 1, while *ϕ* is negative or else it is zero. The label image is converted to probability image *E*_in_ as in equation (30). The curve point at positions with large *E*_in_ should be move outwards, whereas curve point with large *E*_out_ = 1 − *E*_in_ should move inside, and curve must be convergence in band, while *E*_in_ = *E*_out_. The curve evolution is specified as

(32) $$\begin{array}{c} \frac{\partial \phi }{\partial t}=\frac{1}{2}-{Ε}_{\text{in}}+p_{h}\left|\nabla \varphi \right|. \end{array}$$

Every *Χ* labels with single level set function is represented as *ϕ*_*Χ*_, *x* = 1, .⋯, *Χ* for segmenting multiple labels. The pixel-wise transformation of probabilities for every labels are expressed as

(33) $$\begin{array}{c} \left({\tilde{e}}_{1},{\tilde{e}}_{2},\cdots {\tilde{e}}_{Χ}\right)=\left(\begin{array}{ccc} \frac{e_{1}}{e_{1}+\max\left(e_{n}\right)} & \frac{e_{2}}{e_{2}+\max\left(e_{n}\right)},\cdots , & \frac{e_{Χ}}{e_{Χ}+\max\left(e_{n}\right)}\\ n\neq 1 & n\neq 2 & n\neq Χ \end{array}\right). \end{array}$$

Here, the level set evolution for a multilabel segmentation is expressed by the following equation,

(34) $$\begin{array}{c} \frac{\partial \varphi_{x}}{\partial t}=\frac{1}{2}-{\tilde{Ε}}_{x}+p_{h}\left|\nabla \varphi_{x}\right|;x=1,2,\cdots ,Χ. \end{array}$$

Moreover, the curve update is performed by

(35) $$\begin{array}{c} \varphi^{t+1}=\varphi^{t}+\Delta t\frac{\partial \varphi }{\partial t}. \end{array}$$

Based on WCBA, curve update is equation (21) of equation (35),

(36) $$\begin{array}{c} L_{\text{river}}^{b}=\frac{\left(L_{\text{river}}^{b+1}-\mathrm{rand}z\left(L_{\text{river}}^{b-1}+Q^{b-1}\right)\right)\;l_{b}}{1-\mathrm{rand}z\left(2-l_{b}\right)}. \end{array}$$

Substitute equation (36) in equation (35), the solution becomes

(37) $$\begin{array}{c} \varphi^{t+1}=\frac{\left(\varphi^{t+1}-\mathrm{rand}z\left(\varphi^{t-1}+Q^{b-1}\right)\right)\;l_{b}}{1-\mathrm{rand}z\left(2-l_{b}\right)}+\Delta t\frac{\partial \varphi }{\partial t},\\ \varphi^{t+1}=\frac{\varphi^{t+1}\;l_{b}}{1-\mathrm{rand}z\left(2-l_{b}\right)}-\frac{\mathrm{rand}{zl}_{b}\left(\varphi^{t-1}+Q^{b-1}\right)}{1-\mathrm{rand}z\left(2-l_{b}\right)}+\Delta t\frac{\partial \varphi }{\partial t},\\ \varphi^{t+1}-\frac{\varphi^{t+1}\;l_{b}}{1-\mathrm{rand}z\left(2-l_{b}\right)}=\Delta t\frac{\partial \varphi }{\partial t}-\frac{\mathrm{rand}{zl}_{b}\left(\varphi^{t-1}+Q^{b-1}\right)}{1-\mathrm{rand}z\left(2-l_{b}\right)},\\ \varphi^{t+1}\left(1-\frac{l_{b}}{1-\mathrm{rand}z\left(2-l_{b}\right)}\right)=\Delta t\frac{\partial \varphi }{\partial t}-\frac{\mathrm{rand}{zl}_{b}\left(\varphi^{t-1}+Q^{b-1}\right)}{1-\mathrm{rand}z\left(2-l_{b}\right)},\\ \varphi^{t+1}\left(\frac{1-2\mathrm{rand}z+\mathrm{rand}{zl}_{b}-l_{b}}{1-\mathrm{rand}z\left(2-l_{b}\right)}\right)=\Delta t\frac{\partial \varphi }{\partial t}-\frac{\mathrm{rand}{zl}_{b}\left(\varphi^{t-1}+Q^{b-1}\right)}{1-\mathrm{rand}z\left(2-l_{b}\right)},\\ \varphi^{t+1}=\frac{1-\mathrm{rand}z\left(2-l_{b}\right)}{1-\mathrm{rand}z\left(2-l_{b}\right)-l_{b}}\left[\Delta t\frac{\partial \varphi }{\partial t}-\frac{\mathrm{rand}{zl}_{b}\left(\varphi^{t-1}+Q^{b-1}\right)}{1-\mathrm{rand}z\left(2-l_{b}\right)}\right]. \end{array}$$

Therefore, the above expression is utilized for updating the curve; thereby, the proposed WCBA-based deformable model achieved better performance for lung cancer segmentation with less complexities.

## 4. Results and Discussion

The implementation of the proposed work is carried out in Matlab environment. The experiment is conducted using Lung Image Database Consortium image collection (LIDC-IDRI) [24]. This dataset was started by the Foundation for the National Institutes of Health (FNIH). This dataset includes three modalities; lung cancer screening CT scans with annotated lesions, computed radiography, and digital radiography. Moreover, eight medical imaging companies and seven centers are incorporated to produce this database including 1018 cases.

### 4.1. Performance Metrics

The performance metrics which are used to evaluate the proposed WCBA based deformable model approach is as follows.

#### 4.1.1. Average Segmentation Accuracy

Segmentation accuracy is utilized in order to predict the correctness of segmentation and is defined as

(38) $$\begin{array}{c} \text{SegmentationAccuracy}=\frac{A+B}{A+B+K+1}, \end{array}$$

where *A*, *B*, *K*, and *I* denote true positive, true negative, false positive, and false negative, respectively.

#### 4.1.2. Average Jaccard Coefficient

Jaccard coefficient is used to calculate the similarities of two samples. The Jaccard coefficient is expressed as

(39) $$\begin{array}{c} \text{JaccardCoefficient}=\frac{\left|M\cap N\right|}{\left|M\right|+\left|N\right|-\left|M\cap N\right|}, \end{array}$$

where *M* and *N* are samples.

#### 4.1.3. Average Dice Coefficient

This metric is employed to compare segmentation of predicted output and target output.

(40) $$\begin{array}{c} \text{DiceCoefficient}=\frac{2\times \left(Y\cap Z\right)}{\left(Y\cup Z\right)}, \end{array}$$

where *Y* is a target output and *Z* signifies predicted output.

### 4.2. Segmentation Results

**Figure 2:**
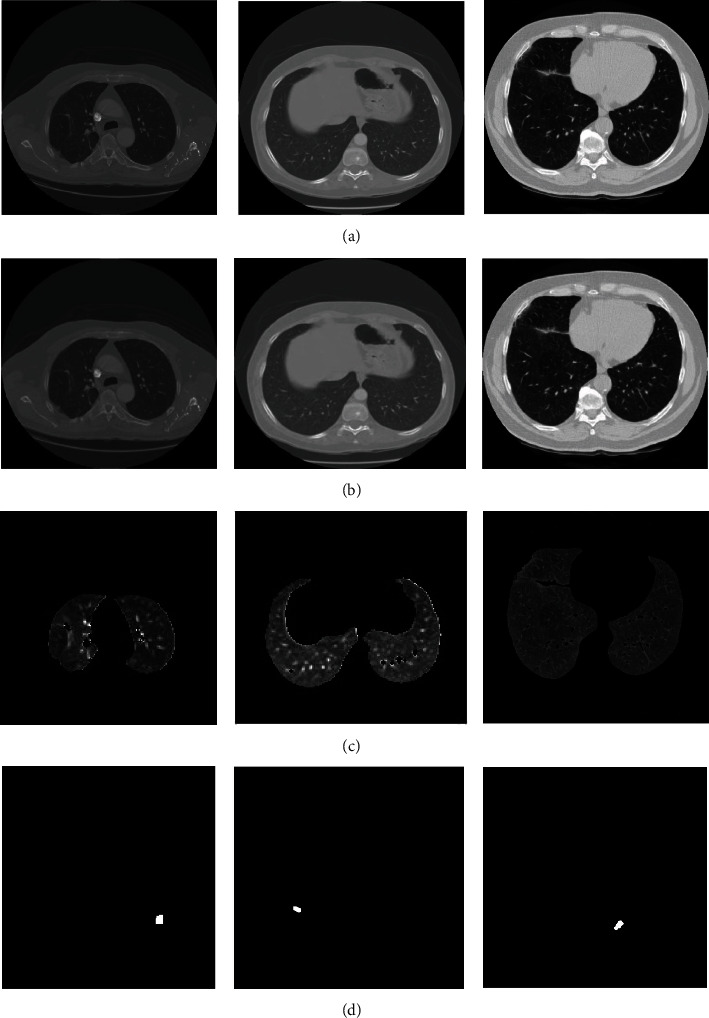
Segmentation results of the proposed method: (a) input images, (b) preprocessed images, (c) lung lobes region segmentation, and (d) lung cancer segmentation.

The experiment is conducted on three sample CT images.  Figure 2 illustrates the segmentation results obtained by the developed WCBA based deformable model.  Figure 2(a) represents three sample images of CT, Figure 2(b) shows preprocessed images, Figure 2(c) depicts the lung lobes regions segmentation, and Figure 2(d) represents lung cancer segmentation. The performance evaluation and comparative analysis of the proposed method are discussed in the following sections.

### 4.3. Performance Evaluation

**Figure 3:**
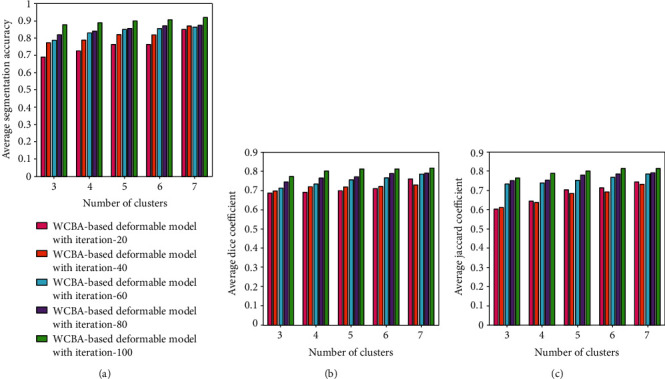
Performance evaluation of the proposed method for different values of iterations: (a) average segmentation accuracy, (b) average Dice coefficient, and (c) average Jaccard coefficient.

The performance of the proposed method is evaluated using three metrics, namely, average segmentation accuracy, average dice coefficient, and average Jaccard coefficient for the cluster size of 7 with different iteration values as depicted in Figure 3. For the cluster size of 7, the average segmentation accuracy of the proposed method is 0.8504, 0.8704, 0.8660, 0.8746, and 0.9205 for the corresponding iterations of 20, 40, 60, 80, and 100 as shown in Figure 3(b). Similarly, the average dice coefficients are 0.7605, 0.7281, 0.7862, 0.7909, and 100 as shown in Figure 3(b), and the average Jaccard coefficient is 0.7429, 0.7304, 0.7844, 0.7913, and 0.8136 as shown in Figure 3(c).

### 4.4. Comparative Analysis

**Figure 4:**
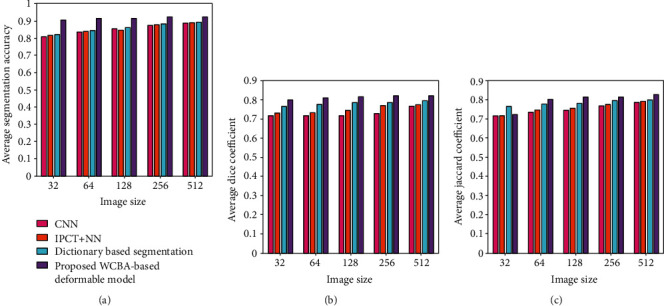
Comparative analysis of the proposed method for the cluster size 4: (a) average segmentation accuracy, (b) average Dice coefficient, and (c) average Jaccard coefficient.

The comparative analysis of the proposed WCBA based deformable model is performed with the existing techniques, namely, CNN [3], IPCT + NN [14], and dictionary-based segmentation [21]. Three evaluation metrics, average segmentation accuracy, average Dice coefficient, and average Jaccard coefficient, are used for the performance comparison with different cluster size as well as image size as depicted in Figure 4. For cluster size 4 and image size 256 × 256, the proposed method achieved an average segmentation accuracy of 0.9245 and correspondingly the other methods achieved 0.8745, 0.8788, and 0.8823 as shown in Figure 4(a).

Here, the proposed method obtained a better percentage improvement of 5.40%, 4.94%, and 4.56% with respect to the existing techniques. Likewise, in Figure 4(b), the average Dice coefficient obtained by the proposed method is 08208 and for other techniques 0.7278, 0.7696, and 0.7863. Hence, the percentage of improvement of the proposed method is 11.31%, 6.23%, and 4.20%. In Figure 4(c), the average Jaccard coefficient of the proposed method is 0.8155 and for the other techniques 0.7657, 0.7753, and 0.7953, respectively. In this case, the percentage of improvement is 6.10%, 4.92%, and 2.47%.

**Figure 5:**
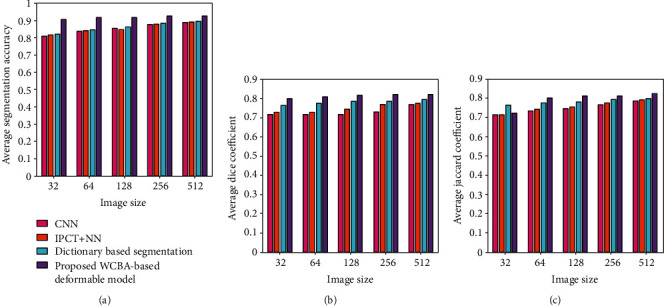
Comparative analysis of the proposed method for the cluster size 5: (a) average segmentation accuracy, (b) average Dice coefficient, and (c) average Jaccard coefficient.

Similarly, the performance comparison o7f the proposed method with the existing techniques is shown in Figure 5 for the cluster size 5 and image size 512 × 512. As depicted in Figure 5(a), the average segmentation accuracy achieved by the proposed method is 0.9024, while other methods achieved 0.8224, 0.8591, and 0.8766, respectively. Here, the percentage improvement of the proposed model is 8.86%, 4.79%, and 2.85% as against to the existing techniques. Furthermore, the average Dice coefficient obtained by the proposed method is 0.8261 and for other methods 0.7282, 0.7832, and 0.7999, respectively, as shown in Figure 5(b). In this case, the improvement obtained by the proposed method is 11.84%, 5.20%, and 3.17%. Likewise, in Figure 5(c), the average Jaccard coefficient obtained by the proposed method is 0.8228 and for other methods 0.7437, 0.7702, and 0.7977, respectively. Here, the proposed method attained the performance improvement of 9.61%, 6.39%, and 3.05%, respectively.  Table 1 summarizes the performance of the proposed method with the existing techniques in terms of average segmentation accuracy, Dice coefficient, and Jaccard coefficient for different cluster and image size.

Tables 2 and 3 illustrate the comparison of computational time and memory utilization of the proposed method and existing techniques. From Table 2, it is observed that the processing speed of the proposed method is high and hence computationally efficient. Similarly, from Table 3, it is noticed that the memory requirements of the proposed method are less as compared to other techniques.

## 5. Conclusion

In this paper, a WCBA-based deformable model for lung cancer segmentation is presented. This approach consists of preprocessing, lung lobe regions segmentation, and lung cancer segmentation. After preprocessing, accurate lung lobe regions are extracted using Bayesian fuzzy clustering models. Once the lung lobe regions are extracted, then, lung cancer segmentation is performed using the WCBA-based deformable model. The proposed WCBA is devised by the incorporation of BA and WCA. The experiment was carried out using standard database. Furthermore, the performance is evaluated using average segmentation accuracy, average Dice coefficient, and average Jaccard coefficient for different cluster sizes. The results obtained by the proposed method are very encouraging as compared to the existing lung cancer segmentation techniques. The future dimension of this work can be extended by developing other novel optimization algorithms for obtaining better segmentation performance.

## Figures and Tables

**Algorithm 1:**
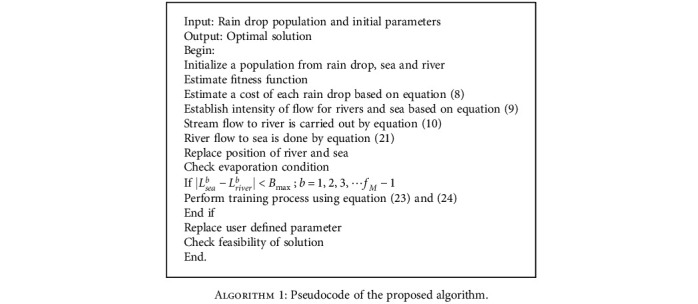
Pseudocode of the proposed algorithm.

**Table 1:** Performance comparison of the proposed method with the existing techniques.

Cluster size	Evaluation metrics	CNN [3]	IPCT + NN [14]	Dictionary-based method [21]	Proposed method
4	Average segmentation accuracy	0.8875	0.8909	0.8934	0.9257
	Average Dice coefficient	0.7667	0.7754	0.7963	0.8213
	Average Jaccard coefficient	0.7855	0.7911	0.7997	0.8244

5	Average segmentation accuracy	0.8424	0.8591	0.8791	0.9257
	Average Dice coefficient	0.7645	0.7899	0.8085	0.8282
	Average Jaccard coefficient	0.7579	0.7844	0.7991	0.8270

**Table 2:** Comparison of computational time.

Methods	CNN [3]	IPCT + NN [14]	Dictionary-based method [21]	Proposed method
Time in sec.	17.971	16.707	14.077	12.582

**Table 3:** Comparison of memory requirements.

Methods	CNN [3]	IPCT + NN [14]	Dictionary-based method [21]	Proposed method
Memory in GB	3.803	3.8003	3.787	3.728

## Data Availability

The codes along with dataset images are available on github repository. Link https://github.com/mamthashetty234/Water-Cycle-Bat-Algorithm.git.
